# The Effect of Metacognitive Knowledge on Mathematics Performance in Self-Regulated Learning Framework—Multiple Mediation of Self-Efficacy and Motivation

**DOI:** 10.3389/fpsyg.2018.02518

**Published:** 2018-12-12

**Authors:** Yi Tian, Yu Fang, Jian Li

**Affiliations:** ^1^Faculty of Psychology, Beijing Normal University, Beijing, China; ^2^Beijing Research Center for Education Supervision and Quality Assessment, Beijing Academy of Educational Sciences, Beijing, China; ^3^Beijing Key Laboratory of Applied Experimental Psychology, Beijing, China

**Keywords:** self-regulated learning, metacognitive knowledge, self-efficacy, motivation, mathematics performance

## Abstract

Metacognition, self-efficacy, and motivation are important components of interaction in self-regulated learning (SRL). However, the psychological mechanism underlying the association among them in mathematical learning remained ambiguous. The present study investigated whether the relationship between metacognitive knowledge (MK) and mathematics performance can be mediated by self-efficacy and motivation. The sample comprised 569 students (245 male, Mage = 16.39, *SD* = 0.63) of Grade 10 in China. The MK in mathematics questionnaire, the self-efficacy questionnaire, the academic motivation scale, Raven advanced progressive matrix, and mathematics tests were used for data collection. Our results suggested that the mathematics performance could be predicted by MK, self-efficacy and intrinsic motivation. Moreover, the association between MK and mathematics performance was mediated by self-efficacy and intrinsic motivation, as revealed by a multiple mediation analysis. Additionally, there were sex differences in MK, self-efficacy and intrinsic motivation. The findings highlight the psychological mechanism in the mathematics of Chinese students and will help teachers to improve students’ mathematical learning in SRL framework more effectively. Implications for education and further studies are discussed.

## Introduction

The question, that how to lead our students into academic successes, has received great research interest for decades. Many crucial psychological constructs underlying effective learning have been proposed such as academic aptitude, motivation, and so on. Self-regulated learning (SRL), which stands on the shoulders of many previous successful psychological constructs, is a powerful construct that distal the critical gradients in effective learning, or development of skills and abilities (Tressel et al., 2018). The SRL concept, firstly proposed by Zimmerman (1986), emphasizes the role of learner in monitoring, controlling, and adjusting the learning process in a positive and conscious manner.

Self-regulated learning is a multidimensional construct that emphasizes the active role of the learner (Greene and Azevedo, 2010; Winne, 2010; Efklides, 2011). The effectiveness of SRL in academic success has been supported by many studies. For example, Caprara et al. (2008) suggested that high perceived efficacy for SRL in junior high school contributed to junior high school grades. Mega et al. (2014) considered SRL as the positive predictor of academic achievement. Thus, SRL has become one of the most important research areas in educational psychology, and many researchers proposed their theoretical model for the general learning. Panadero (2017) analyzed and compared six models of SRL, and concluded that SRL included the cognitive, metacognitive, behavioral, motivational, and emotional/affective aspects of learning. Comparing these models, Efklides (2011) model – Metacognitive and Affective Model of SRL (MASRL) had a stronger metacognitive background, and motivation and affect occupy a central role in Efklides’ figure. Although the model has been gained successes in accounting for some educational domains (Clark, 2012; Panadero and Jonsson, 2013; Mega et al., 2014; Stylianou-Georgiou and Papanastasiou, 2017; Baars and Wijnia, 2018), it is still ambiguous whether the model have power to predict mathematics achievement. Because of the importance of mathematical education around the world, especially in China (Yan, 1994), the present research chose the mathematics subject to explore how aspects in SRL influenced the mathematics performance.

### Metacognitive Knowledge and Mathematics Performance

Metacognition plays an important role in models of SRL (Borkowski et al., 2000; Puustinen and Pulkkinen, 2001) and in academic performance (Zohar and Peled, 2008; Harris, 2015). The role of metacognition in several domains such as mathematics (De et al., 2000; Desoete et al., 2001, 2003; Callan and Cleary, 2018), reading competence (Soodla et al., 2016), language learning (Wang and Han, 2017), and even music (Barbara and Alessandro, 2017) has been advocated.

Recent studies discussed metacognition under three main components: Metacognitive knowledge (Flavell, 1979; Efklides, 2001), metacognitive control (Brown, 1980; Desoete and Roeyers, 2006; Sungur, 2007), and metacognitive experiences (Flavell, 1979; Efklides, 2008). Metacognitive control and experiences are considered metacognitive processes, also referred to as online metacognition (Desoete, 2008). The importance of the two components of metacognition in learning mathematics has been abundantly demonstrated (Efklides et al., 1998; Efklides, 2001; Veenman, 2006; Roebers et al., 2012, 2014; Tornare et al., 2015), but metacognitive knowledge, which forms the knowledge subcomponent of metacognition (Flavell, 1979) and consists of self, task, and strategy knowledge (Efklides, 2001), only has received less attention in mathematical learning research (Neuenhaus et al., 2011). Especially in china, there were more rarely empirical studies about metacognitive knowledge in mathematics (Tang and He, 2009). Meanwhile, metacognitive knowledge relevant for school-related domains can be effectively trained in the secondary school (Schneider, 2008). So for the present study, we chose to investigate the metacognitive knowledge as the predictor of mathematics performance.

For mathematics, metacognitive knowledge refers to the mathematical processes and techniques students have and their ideas about the nature of mathematics (Özsoy, 2011). In order to measure metacognitive knowledge in mathematics, Efklides and Vlachopoulos (2012) developed the Metacognitive Knowledge in Mathematics Questionnaire (MKMQ). Desoete et al. (2001) indicated that metacognitive knowledge and skills accounted for 37% of the performance in mathematical problem solving. Some researches found MK was positively correlated with the mathematic performances (Desoete et al., 2001; Schneider and Artelt, 2010; Özsoy, 2011).

### Metacognitive Knowledge, Self-Efficacy, Motivation, and Mathematics Performance

Prior research has confirmed that metacognitive knowledge is related to mathematics performance (Desoete et al., 2001). Meanwhile, a large number of studies found that self-efficacy and intrinsic motivation, respectively, had positive correlation with students’ mathematics performances (Stevens et al., 2004; Chen, 2010; Van Slooten, 2013; Briggs, 2014). At the same time, there had been plenty of studies discussing the relationship between self-efficacy and intrinsic motivation, which indicated that higher self-efficacy can lead to higher intrinsic motivation (Bandura et al., 1999; Matosic et al., 2014; You et al., 2016; Ariani, 2017). Besides, empirical studies on self-efficacy and motivation showed that self-efficacy in mathematical learning could affect the students’ motivated actions like efforts, persistence, and seeking for help through intrinsic motivation in mathematical learning (Skaalvik et al., 2015). Taking all those results into account, we may generate an idea to explore whether MK exerts an indirect effect on mathematics performance through both self-efficacy and intrinsic motivation. We proposed this hypothesis according to the following logic.

First, some researches showed that promoting metacognitive and strategic knowledge would enhance the learners’ self-efficacy (Liu, 1998). Efklides (2011) pointed out that MK of self and self-efficacy was positive interrelated. Sang and Wang (2001) suggested that MK of self mainly affected on the students’ self-efficacy while describing the effect of MK on learning. Further study showed that the relationship between metacognition and performance was fully mediated by self-efficacy (Coutinho, 2008). Second, the empirical study of Carr et al. (1994) found out that MK and motivation were significantly positive correlated. Researchers demonstrated the mutual effects between MK of strategies and motivation (Borkowski et al., 2000). In addition, MK of strategies was believed to play positive role in students’ academic motivation (Paris and Paris, 2001). Meanwhile, intrinsic motivation as a part of academic motivation plays more important role with regard to school achievement because of its inherent relationship with cognitive processing (Gottfried, 1985). Some contemporary theories incorporate intrinsic motivation in their formulations. For example, in self-determination theory, intrinsic motivation is presented as the prototype of autonomous and self-determined behavior (Deci and Ryan, 2000). Besides, some empirical studies on self-efficacy and motivation showed that self-efficacy in mathematical learning could affect the students’ motivated actions like efforts, persistence, and seeking for help through intrinsic motivation in mathematical learning (Skaalvik et al., 2015).

### Sex Difference in Mathematics Performance

Sex differences in mathematics performance also need to be paided attention. Researchers found that male students were better at mathematics performance, especially when dealing with higher cognitive applications (Royer et al., 1999; Casey et al., 2001). However, there is still evidence that the sex differences are weakening or even disappearing. Hyde et al. (2008) obtained 7 million American children’s data from a selection of 10 states across Grades 2–11 and found no gender difference in math performance. Scafidi and Bui (2010) replicated the findings, and showed gender similarities in performance on standardized math tests in Grade 8, 10, and 12. Some studies even showed that male students scored significantly lower in mathematical problem solving than female students (Stoel et al., 2003). A recent study on Chinese students demonstrated that there was no gender differences in Grade 5, and a relatively small gender differences emerging in Grade 8 with females scored higher than males (Li et al., 2017). The nature of sex difference is ambiguous. Recent studies focus on the ability to exploit their metacognitive knowledge (Schneider and Artelt, 2010), different self-concept between boys and girls (Kling et al., 1999), or intrinsic motivation (Freudenthaler et al., 2010; Steinmayr and Spinath, 2010).

### Current Study

**FIGURE 1:**
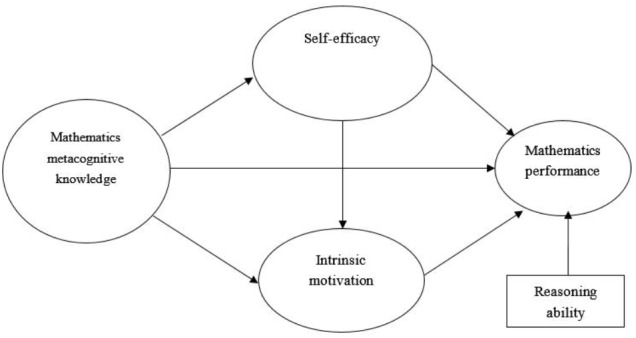
The hypothesized model of MK, self-efficacy, intrinsic motivation, and mathematics performance.

The present study investigated the concurrent mediation effect of self-efficacy and motivation on the relationship between metacognitive knowledge and mathematics performance. We expected to build a self-regulated model of mathematical learning on the basis of previous studies of SRL framework, and analyzed the sex differences of mathematics performance, MK, self-efficacy, and motivation. Figure 1 shows the hypothesized model of the present study. Since intelligence must be considered when estimating metacognition’s ability to predict academic performance (Ohtani and Hisasaka, 2018), and mathematical reasoning ability could reliably predict success in mathematics attainment (Adegoke, 2013), we brought reasoning ability representing intelligence into our model as a controlled variable.

We hypothesized that: (1) MK could positively predict mathematics performance. (2) Self-efficacy mediated the association between MK and mathematics performance. (3) Motivation mediated the relationship between MK and mathematics performance. (4) MK exerts a significant indirect effect on mathematics performance through the three-path mediating effect of self-efficacy and motivation. (5) Male students were significantly higher in mathematics performance, MK, self-efficacy, and motivation than female students.

## Materials and Methods

### Participants

A total of 612 students in 10th grade from an ordinary high school participated in the present study. The research protocol was approved by the IRB of the Faculty of Psychology, BNU. Accordingly, prior to participation, students were informed about the general aim of the research and the anonymity of their data. Participation was voluntary, and students received small gift like stationery for their participation in the study. Informed consent forms were collected from the students’ parents. To control the response quality, two items (*“please choose the answer of 2 plus 0” “please choose 4 in this item”*) were contained in the questionnaire. If one chose the wrong answers in both, he/she would be excluded. Finally, 43 students were excluded. Final analysis was carried out on 569 students (324 female and 245 male, mean age = 16.39 years, *SD* = 0.63).

### Measures

#### Metacognitive Knowledge in Mathematics

Metacognitive Knowledge in Mathematics Questionnaire is a useful instrument for depicting people’s beliefs about themselves as processors of mathematical tasks (Efklides and Vlachopoulos, 2012). MKMQ consists of seven interrelated subscales, which are categorized into three domains:

(a) Metacognitive knowledge of self. Participants need to judge whether the following statements are in accordance with their situation on a 5-point Likert scale from 1 (not at all true of me) to 5 (absolutely true of me). MK of the Self (easiness/fluency) subscale consists of six items like “I immediately understand mathematical problems whatever they require,” while MK of the Self (difficulty/lack of fluency) subscale contains seven items like “When I read a problem with many words I do not understand what I have to do.”
(b) Metacognitive knowledge of tasks. Participants are required to judge how difficult are the following items on a 5-point Likert scale from 1 (not difficult) to 5 (very difficult). Subscale of MK of Tasks (easy/low demands) consists of six items like “Requires division,” while subscale of MK of Tasks (difficult/high demands) compromises five items like “Has many operations.”
(c) Metacognitive knowledge of strategies. Participants are expected to answer if they often come across following situation based on their real circumstances on a 5-point Likert scale from 1 (never) to 5 (always). Subscale of MK of strategies (cognitive/metacognitive) comprises 10 items like “When I have solved a mathematical problem I am checking if I did the computations correctly,” and subscale of MK of strategies (competence-enhancing strategies) includes five items like “When I learn something new in mathematics I am checking how it is connected to previous lessons,” while subscale of MK of strategies (avoidance strategies) consists of six items like “When I do not understand what the mathematical problem requires I give up.”

The MKMQ was translated into Chinese, blindly back-translated into English and then translated into Chinese again by two researchers proficient in Chinese and English to avoid any potential misunderstandings and ensure translation accuracy. Some slight modifications in phrasing were made to make items more appropriate for Chinese students. Reliabilities for each scale/subscale score in this study were presented in Table 1.

**Table 1:** Sample items and test score reliability.

Scale/subscale	n	Sample items	ω (CI)	CR (CI)
MK of self (easiness/fluency)	6	I immediately understand mathematical problems whatever they require	0.82 (0.79, 0.85)	0.83 (0.81, 0.85)
MK of self (difficulty/lack of fluency)	7	When I read a problem with many words I do not understand what I have to do	0.76 (0.71, 0.79)	0.76 (0.72, 0.79)
MK of tasks (easy task)	6	Requires division	0.89 (0.87, 0.91)	0.89 (0.87, 0.91)
MK of tasks (difficult task)	5	Has many operations	0.82 (0.80, 0.85)	0.83 (0.80, 0.85)
MK of strategies (cognitive/metacognitive strategies)	10	When I have solved a mathematical problem I am checking if I did the computations correctly	0.82 (0.79, 0.85)	0.83 (0.81, 0.85)
MK of strategies (competence-enhancing strategies)	5	When I learn something new in mathematics I am checking how it is connected to previous lessons	0.71 (0.66, 0.76)	0.71 (0.66, 0.75)
MK of strategies (avoidance strategies)	6	When I do not understand what the mathematical problem requires I give up	0.73 (0.68, 0.77)	0.73 (0.68, 0.76)
Intrinsic motivation	12		0.70 (0.68, 0.72)	0.93 (0.92, 0.94)
To know	4	Because mathematical study allows me to continue to learn about many things that interest me	0.85 (0.82, 0.87)	0.85 (0.82, 0.87)
To accomplish	4	For the satisfaction I feel when I am in the process of accomplishing difficult mathematical problems	0.85 (0.81, 0.88)	0.85 (0.81, 0.87)
To experience stimulation	4	Because I really like learning mathematics	0.83 (0.80, 0.86)	0.84 (0.82, 0.87)
Self-efficacy	5	I am certain that I can do excellent job on problems and tasks assigned for mathematics class	0.83 (0.79, 0.85)	0.84 (0.81, 0.86)
RAPM	32	N/A	0.74 (0.68, 0.79)	0.78 (0.73, 0.82)
Mathematics performances	3	N/A	0.85 (0.82, 0.87)	0.85 (0.82, 0.87)


*n, number of items; ω (CI), omega as an index of reliability with 95% CI; CR (CI), composite reliability with 95% CI.*

#### Self-Efficacy in Mathematics

The five items Self-efficacy Questionnaire was chosen and adapted to assess participants’ self-efficacy in mathematics (Bong, 2001). We revised the original statements with mathematic learning situations, like “I am certain that I can do excellent job on problems and tasks assigned for mathematics class.” Participants rated how those statements apply to them on a 5-point Likert-type scale. A higher score indicates higher level of self-efficacy in mathematics.

#### Intrinsic Motivation of Mathematical Learning

The Chinese version of Academic Motivation Scale (CAMS) was chosen and adapted to assess participants’ mathematical learning motivation (Bo et al., 2016). The CAMS contains seven subscales, which are amotivation (AM), external regulation (EMER), introjected regulation (EMIN), identified regulation (EMID), intrinsic motivation to know (IMTK), intrinsic motivation to accomplish (IMAC), and intrinsic motivation to experience stimulation (IMTE). Each subscale contains four items. We chose and revised three subscales, which were IMTK, IMAC, and IMTE, as the measurement of intrinsic motivation of mathematical learning. For example, the subscale IMTK contains items like “Because mathematical study allows me to continue to learn about many things that interest me,” the subscale IMAC contains items like “For the satisfaction I feel when I am in the process of accomplishing difficult mathematical problems,” the subscale IMTE contains items like “Because I really like learning mathematics.” Participants rated how those statements apply to them on a seven-point Likert-type scale. A higher score indicates higher level of intrinsic motivation of mathematical learning.

#### Raven Advanced Progressive Matrix (RAPM)

Raven Advanced Progressive Matrix was used to assess students’ reasoning ability (Raven et al., 1998). There are 36 items and the maximum score is 36. Each item consists of a 3 × 3 matrix of which the right lower element is missing. Participants need to determine how elements change and select the correct element from eight options to complete the matrix. The total score would be used in data analysis.

#### Mathematics Performance

The scores of three successive mathematics examinations, which took place after the administration of the test battery, were collected by the school coordinators as the measurement of participants’ mathematics performance.

### Procedure

School coordinators arranged the time for all participants to complete the test battery. Each class was equipped with a trained research assistant. Standardized instruction about the purpose of the study was delivered first. Students were reminded to complete the battery all by themselves. The head teacher of each class was also present to keep order. When the students finished all questionnaires, they would take a 10 min’ break, and then began to answer the RAPM within 40 min. After that, students handed in the battery directly to the research assistant.

### Data Analyses

As presented in the section “Results,” data analysis began with descriptive statistics for all measures and their inter-correlations were computed for examination and reporting. Next, five multivariate analysis of variance (MANOVA) were performed to test for mean differences. In the first, the two factors of MK of self were considered as dependent variables and sex as independent variable. In the second, the two factors of MK of tasks were the dependent variables, while in the third, the three factors of MK of strategies were the dependent variables, and in the fourth, the three factors of intrinsic motivation were the dependent variables, and in the fifth, three mathematic performances were the dependent variables, whereas the independent variables remained unchanged. In addition, two univariate analysis of variance (ANOVA) were performed to test for mean differences between male and female in self-efficacy in mathematics and reasoning ability, respectively. Descriptive statistics and correlation analysis were conducted using IBM SPSS 20 (IBM, 2012).

In the second step, multiple mediation (Preacher and Hayes, 2008) with 10 latent variables in the structural equation modeling (SEM) approach (Kline, 2011) was conducted to examine self-efficacy and intrinsic motivation as potential mediators of the association between seven metacognitive knowledge factors and mathematics performance. Reasoning ability was entered as a control variable. Confirmatory factor analysis (CFA) and SEM were conducted using Mplus 7.0 (Muthén and Muthén, 1998/2017).

When estimating the indirect effect, Sobel test, which is also called the product-of-coefficients approach, is one of the most commonly used methods (Sobel, 1982, 1986). However, Sobel test is not recommended by researchers recently, because the assumption of normality of the sampling distribution is typically not satisfied (Montoya and Hayes, 2017). Other approaches, for example, bootstrap method, is more recommended and widely used for its better performance (Mackinnon et al., 2004). Bootstrapping works by repeatedly sampling from the data set for thousands of times, which helps estimating the sampling distribution of ab. Afterward, the confidence intervals (CIs) for the indirect effect are constructed (Preacher and Hayes, 2008). As noted in simulation studies (Mackinnon et al., 2002), the bias-corrected bootstrap performs best with higher power and reasonable control over the Type I error rate. This approach is especially recommended for multiple mediator models (Preacher and Hayes, 2008). We used a 95% CIs method in the present study to determine the significance of these indirect effects. According to this approach, the indirect effect is significant if the CI does not include zero. All structural models were evaluated using fit indices following Kline (2011) recommendations. We used Root Mean Square Error Approximation (*RMSEA*), Standardized Root Mean Square Residual (*SRMR*), the Comparative Fit Index (*CFI*), and the Tucker–Lewis Index (*TLI*) as well as the general fit based on χ^2^ test of model fit. We used the most widely recommended cut-off values indicative of an adequate model fit to the data, respectively: χ^2^/*df* < 3 (Kline, 2011), RMSEA, and SRMR < 0.06 and <0.08, CFI and TLI > 0.90 (Browne and Cudeck, 1992).

## Results

### Descriptive Statistics and Correlations Between the Measured Variables

Correlations, means, and standard deviations of the measures are presented in Table 2. Preliminary speculation on correlations among variables indicated that MK of self (easiness/fluency), MK of strategies (cognitive/metacognitive strategies), and MK of strategies (competence-enhancing strategies) were positively related to intrinsic motivation, self-efficacy in mathematics, and mathematics performances. However, MK of self (difficulty/lack of fluency), MK of tasks (easy task), MK of tasks (difficult task), and MK of strategies (avoidance strategies) were negatively related to intrinsic motivation, self-efficacy in mathematics, and mathematics performances. We also found significant positive correlation between mathematics performances and intrinsic motivation, as well as self-efficacy in mathematics. These results suggested a potential indirect effect from MK to mathematics performance. Significant correlations were found between reasoning ability and all three mathematics performances, which encouraged us to bring reasoning ability into the mediation model.

**Table 2:** Correlations between the measured variables, scale/subscale means, and standard deviations for male and female participants.

	F1	F2	F3	F4	F5	F6	F7	F8	F9	F10	F11	F12	F13	F14	F15
F2	-0.49**														
F3	-0.32**	0.37**													
F4	-0.41**	0.48**	0.45**												
F5	0.44**	-0.40**	-0.23**	-0.34**											
F6	0.33**	-0.22**	-0.06	-0.20**	0.53**										
F7	-0.29**	0.30**	0.15**	0.31**	-0.21**	-0.14**									
F8	0.35**	-0.28**	-0.16**	-0.29**	0.39**	0.41**	-0.29**								
F9	0.33**	-0.25**	-0.18**	-0.25**	0.42**	0.35**	-0.23**	0.78**							
F10	0.38**	-0.29**	-0.12**	-0.30**	0.40**	0.40**	-0.35**	0.78**	0.69						
F11	0.54**	-0.44**	-0.23**	-0.33**	0.49**	0.39**	-0.29**	0.46**	0.43**	0.49**					
F12	0.17**	-0.09**	0.01	-0.07	0.07	0.10*	-0.03	-0.01	0.03	0.03	0.08				
F13	0.27**	-0.20**	-0.05	-0.18**	0.28**	0.22**	-0.16**	0.28**	0.31**	0.40**	0.35**	0.22**			
F14	0.21**	-0.20**	-0.06	-0.20**	0.26**	0.16**	-0.21**	0.29**	0.33**	0.40**	0.33**	0.13**	0.68**		
F15	0.19**	-0.14**	-0.06	-0.17**	0.26**	0.17**	-0.15**	0.29**	0.32**	0.37**	0.32**	0.12**	0.61**	0.63**	
MMal	19.51	15.98	8.42	12.51	28.44	10.86	15.03	19.10	20.18	16.42	16.60	23.52	90.98	65.49	84.45
*SD*	4.97	5.1	3.76	3.73	7.06	3.41	4.05	5.41	7.48	5.98	4.34	4.88	27.60	25.02	14.73
MFel	16.64	16.51	8.84	13.41	26.51	9.81	16.34	17.83	19.25	14.76	14.56	23.28	80.95	58.61	79.60
*SD*	4.47	4.89	3.82	4.21	3.41	2.98	4.24	5.72	5.36	5.96	4.47	4.86	25.01	23.11	12.86


*F1, MK of self (easiness/fluency); F2, MK of self (difficulty/lack of fluency); F3, MK of tasks (easy task); F4, MK of tasks (difficult task); F5, MK of strategies (cognitive/metacognitive strategies); F6, MK of strategies (competence-enhancing strategies); F7, MK of strategies (avoidance strategies); F8, intrinsic motivation to know (IMTK); F9, intrinsic motivation to accomplish (IMAC); F10, intrinsic motivation to experience stimulation (IMTE); F11, self-efficacy in mathematics; F12, reasoning ability; F13–F15, mathematics performances; MMal, mean of male; SD_*M*_, standard deviation of male; MFel, mean of female; SD_*F*_, standard deviation of female. ^∗^*p* < 0.05; ^∗∗^*p* < 0.01.*

### Sex Differences in the Measured Variables

Tests of multivariate analysis of variance (MANOVA) were applied by sex to verify mean differences in MK of self, MK of tasks, MK of strategies, intrinsic motivation, and mathematic performances, respectively. Table 3 shows the results of MANOVA. There are significant differences within sex subgroups for all dependent variables.

**Table 3:** Tests of multivariate analysis of variance (MANOVA).

Dependent variables	Wilks’ lambda	*F*	*df*	Error *df*	*p*	$\eta_{p}^{2}$
MK of self	0.903	28.944	2	538	0.000^∗∗^	0.097
MK of tasks	0.987	3.650	2	559	0.027^∗^	0.013
MK of strategies	0.957	7.942	3	533	0.000^∗∗^	0.043
Intrinsic motivation	0.981	3.541	3	546	0.015^∗^	0.019
Mathematics performances	0.959	7.849	3	555	0.000^∗∗^	0.041


*Independent variable: sex; dependent variables: MK of self (easiness/fluency and difficulty/lack of fluency), MK of tasks (easy task and difficult task), MK of strategies (cognitive/metacognitive strategies, competence-enhancing strategies and avoidance strategies), intrinsic motivation (IMTK, IMAC, and IMTE), mathematics performances (three mathematics examinations). ^∗^*p* < 0.05; ^∗∗^*p* < 0.01. $\eta_{p}^{2}$, effect size for analysis of variance, three levels of effect size are: small ($\eta_{p}^{2}$ = 0.01), medium ($\eta_{p}^{2}$ = 0.06), and large ($\eta_{p}^{2}$ = 0.14).*

Further tests of between-subjects effects found that male students scored higher than female students in MK of self (easiness/fluency) (*F*_(1,539)_ = 52.590, *p* < 0.01, $\eta_{p}^{2}$ = 0.089), MK of strategies (cognitive/metacognitive strategies) (*F*_(1,535)_ = 10.409, *p* < 0.01, $\eta_{p}^{2}$= 0.019), MK of strategies (competence-enhancing strategies) (*F*_(1,535)_ = 14.199, *p* < 0.01, $\eta_{p}^{2}$ = 0.026), IMTK (*F*_(1,48)_ = 6.961, *p* < 0.05, $\eta_{p}^{2}$= 0.013), IMTE (*F*_(1,548)_ = 10.351, *p* < 0.01, $\eta_{p}^{2}$ = 0.019), mathematics performance 1 (*F*_(1,557)_ = 20.188, *p* < 0.01, $\eta_{p}^{2}$ = 0.035), mathematics performance 2 (*F*_(1,557)_ = 11.314, *p* < 0.01, $\eta_{p}^{2}$ = 0.020), and mathematics performance 3 (*F*_(1,557)_ = 17.154, *p* < 0.01, $\eta_{p}^{2}$ = 0.030). While female students scored higher than male students in MK of tasks (difficult task) (*F*_(1,560)_ = 7.312, *p* < 0.01, $\eta_{p}^{2}$ = 0.013), and MK of strategies (avoidance strategies) (*F*_(1,535)_ = 11.199, *p* < 0.01, $\eta_{p}^{2}$ = 0.021). We didn’t found significant sex differences in MK of self (difficulty/lack of fluency), MK of tasks (easy task), and IMAC.

Tests of ANOVA were applied by sex to verify mean differences in self-efficacy in mathematics and reasoning ability. Results indicated a significant difference for self-efficacy (*F*_(1,563)_ = 29.541, *p* < 0.01, $\eta_{p}^{2}$ = 0.050) but not for reasoning ability.

### Multiple Mediators Model With Self-Efficacy and Motivation as Mediators

#### Evaluation of the Model

Confirmatory factor analysis was first conducted to examine the goodness-of-fit of the overall measurement model, in which MK of self (easiness/fluency), MK of self (difficulty/lack of fluency), MK of tasks (easy task), MK of tasks (difficult task), MK of strategies (cognitive/metacognitive strategies), MK of strategies (competence-enhancing strategies), MK of strategies (avoidance strategies), IMTK, IMAC, IMTE, self-efficacy in mathematics, and mathematics performance were measured by 6, 7, 6, 5, 10, 5, 6, 4, 4, 4, 5, and 3 items, respectively. The results showed satisfactory fit indices with χ^2^/*df* = 2.10, CFI = 0.87, TLI = 0.86, RMSEA = 0.05, SRMR = 0.06. The average variances extracted (AVE) was 0.46, and loadings of all items ranging from 0.32 to 0.87. The results of CFA suggested good structural validity of the test scores.

Then item parceling approach was adopted to reduce the complexity of the multiple mediation model and the standard errors of estimated structure (Bandalos, 2002; Little et al., 2002; Nasser and Takahashi, 2003; Bandalos and Leite, 2006). Parceling, as a psychometrics measurement practice, refers to aggregating single items into one or more “parcels” and using these parcel(s) as the indicator(s) of the target latent construct (Little et al., 2002; Nasser and Takahashi, 2003; Matsunaga, 2008). In the present study, each of the seven MK subscales was parceled into two parcels. For the subscale with *N* items, parcel 1 was composed of the first *N/2* (N was an even number) or (*N*+1)/2(*N* was an odd number) items, while parcel 2 was composed of the rest of items. Besides, each subscale of intrinsic motivation was parceled into one parcel, respectively, and these three parcels loaded on a latent variable named intrinsic motivation.

**FIGURE 2:**
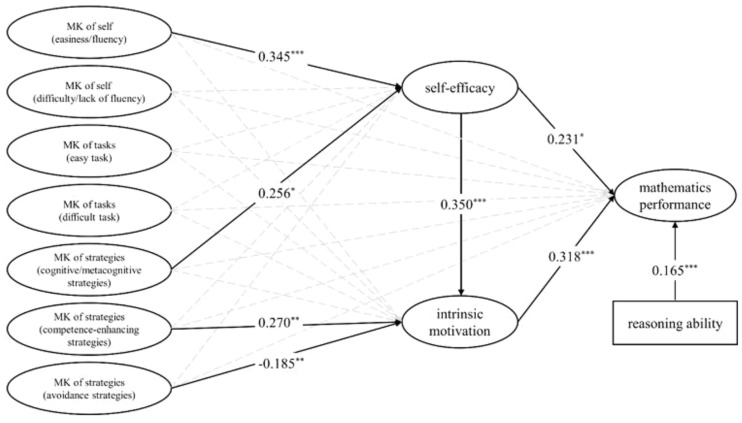
The mediation model for metacognitive knowledge in mathematics on mathematics performance through self-efficacy and intrinsic motivation. Significant paths are in bold lines with standardized b-estimates. ^∗^*p* < 0.05; ^∗∗^*p* < 0.01; ^∗∗∗^*p* < 0.001.

Results indicated that the model with self-efficacy and intrinsic motivation as mediators (Figure 2) achieved quite a good fit to the data, and the AVE was 0.62. The inspection of the fit indices values presented a good fit with χ^2^/*df* = 2.58, CFI = 0.94, TLI = 0.92, RMSEA = 0.05, SRMR = 0.04. The model explained 26.7% of mathematics performances, 23.3% of self-efficacy, and 24.5% of intrinsic motivation.

Data indicate that most of the relationships between the variables is consistent with the hypotheses. First, self-efficacy (β = 0.231, *p* < 0.05) and intrinsic motivation (β = 0.318, *p* < 0.001) were significant predictors of mathematics performance. Besides, self-efficacy could positively predict intrinsic motivation (β = 0.350, *p* < 0.001). Second, MK of self (easiness/fluency) (β = 0.345, *p* < 0.001) and MK of strategies (cognitive/metacognitive strategies) (β = 0.345, *p* < 0.05) could positively predict self-efficacy, while MK of strategies (competence-enhancing strategies) (β = 0.270, *p* < 0.01) and MK of strategies (avoidance strategies) (*β* = -0.185, *p* < 0.01) could predict intrinsic motivation. However, we didn’t found any direct effect between metacognitive knowledge and mathematics performance.

### Indirect Effects of Self-Efficacy and Intrinsic Motivation as Mediators

The indirect effects of seven metacognitive knowledge subscales on mathematics performance by self-efficacy and intrinsic motivation were assessed using multiple mediation models (Preacher and Hayes, 2008) with 95% CIs. Three models with indirect effects were evaluated: (1) seven metacognitive knowledge subscales on mathematics performance mediated only by self-efficacy, (2) seven metacognitive knowledge subscales on mathematics performance mediated only by intrinsic motivation, (3) seven metacognitive knowledge subscales on mathematics performance mediated by sequence of both mediators: self-efficacy and intrinsic motivation. Three parallel mediations and one sequentially two mediations were revealed as significant: parallel mediation of MK of strategies (competence-enhancing strategies) [β = 0.086, *SE* = 0.038, *p* < 0.05, CI (0.012, 0.160)] and MK of strategies (avoidance strategies) [β = -0.059, *SE* = 0.023, *p* < 0.05, CI (-0.104, -0.013)] on mathematics performance by intrinsic motivation; parallel mediation of MK of self (easiness/fluency) [β = 0.080, *SE* = 0.037, *p* < 0.05, CI (0.007, 0.152)] on mathematics performance by self-efficacy; sequential mediation of MK of self (easiness/fluency) on mathematics performance through both self-efficacy and intrinsic motivation [β = 0.038, *SE* = 0.016, *p* < 0.05, CI (0.008, 0.069)].

## Discussion

The present study explored the psychological mechanism in mathematics and investigated the effects of metacognitive knowledge on mathematics performance by self-efficacy and intrinsic motivation in the Chinese context. Results found that metacognitive knowledge had a significant influence on the mathematics performance by the mediation of self-efficacy and intrinsic motivation. Our results confirmed some results from previous studies and also established new relationships among these variables.

In the model, MK could not directly predict mathematics performance, and this was inconsistent with hypothesis 1. However, MK could significantly predict mathematics performance by the mediation of self-efficacy and intrinsic motivation.

Self-efficacy is intricately connected to MK from the point of view of metacognition (Efklides, 2011). Previous research showed that promoting metacognitive and strategic knowledge would enhance the learners’ self-efficacy (Liu, 1998). Our results demonstrated that self-efficacy mediated the association between MK of self (easiness/fluency) with mathematics performance. Therefore, hypothesis 2 was partly supported.

The present study found that MK of strategies (cognition/metacognition) had positive relationship with self-efficacy, while MK of strategies (competence-enhancing strategies) and MK of strategies (avoidance strategies) had positive relationship with intrinsic motivation. The similar result was reached by Karlen et al. (2014). Further analysis showed that MK of strategies (competence-enhancing strategies) and MK of strategies (avoidance strategies) could predicted mathematics performance through the mediation of intrinsic motivation, which is similar to the findings in previous researches (Borkowski et al., 2000). Therefore, hypothesis 3 was partly supported. These results supported and expanded MASRL, which suggested that metacognitive knowledge, skills, and motivation were interrelated and interacted on each other in mathematics field (Efklides, 2011). According to theories of SRL, knowledge about relevant metacognitive strategies improves comprehension only when learners are also motivated to use these strategies (Maier and Richter, 2014). So in mathematics teaching, the teacher should pay special attention to students’ intrinsic motivation of mathematical learning, which is useful for them to adopt MK of strategies more actively and effectively.

Meanwhile, it is interesting to find that the path of MK of self (easiness/fluency) to self-efficacy to motivation to mathematics performance was significant. This path indicated that students who felt very easiness or fluency in mathematical learning had high self-efficacy, which would use their feeling better to facilitate their motivation like efforts, persistence and seeking for help through intrinsic motivation in mathematical learning (Skaalvik et al., 2015), and in turn, may lead to high mathematics performance (Cerasoli et al., 2014). In other words, self-efficacy is a mediator between MK of self (easiness/fluency) and intrinsic motivation, and intrinsic motivation is a mediator between self-efficacy and mathematics performance. Self-efficacy beliefs constitute a powerful motivational factor in SRL (Pintrich, 2000). The hypothesized sequential mediation effects of self-efficacy and intrinsic motivation between MK and mathematics performance were partially supported in our study.

Contrary to our expectations, results found that MK of tasks (easy tasks) and MK of tasks (difficult tasks) had low correlation with self-efficacy, motivation, and mathematics performance, while MK of self (difficulty/lack of fluency) had no influence on the mathematics performance. These results did not fit previous findings which showed that MK of persons and tasks were implicated in motivation in the sense of creating expectations of success (Efklides, 2011). Possibly, this difference can be explained by the item description of the MKMQ. In the current study, MK of self (difficulty/lack of fluency) was measured using these items like “I do not understand the fractions very well” or “I often make mistakes when solving problems with decimals,” and the MK of tasks tapped task demands, that is, easy/low demands mathematical tasks (e.g., subtraction, division, multiplication, addition) versus difficult/high demands mathematical tasks (e.g., fractions, decimals) (Efklides and Vlachopoulos, 2012). However, Chinese mathematics education might benefit from a solid foundation. In China, students are required to practice frequently in those difficult demands mathematics task, which is a basic skill training (Zhang, 2015). So most of students might feel easy for these tasks in China.

As hypothesized, male students had higher mathematics performance than female students, but male students and female students had different advantage on metacognitive knowledge. For example, male students scored higher than female students in MK of self (easiness/fluency), female students scored higher than male students in MK of tasks (difficult task). This finding seems to indicate that girls do not make full use of MK of tasks (difficult task) in solving mathematical problems, which expanded the previous research (Schneider and Artelt, 2010). Meanwhile, male students had significantly higher self-efficacy and intrinsic motivation than female students, which were consistent with the prior research result (Joët et al., 2011; Lee and Kim, 2014). However, there were also several studies showing no sex difference in self-efficacy and intrinsic motivation (Parameswari and Shamala, 2012; Lau et al., 2018).

Our findings have practical implications for educational settings. Regarding students’ mathematics achievement, a slightly greater improvement was found for the students with SRL training (Leidinger and Perels, 2012). The metacognitive training positively affects mathematical problem solving (Lucangeli et al., 1995). Concerning the psychological mechanism of mathematical learning in the SRL framework, we could take more effective measurement to carry out metacognitive training. Improving self-efficacy could be helpful to students’ mathematic learning (Ayotola and Adedeji, 2009). These findings lend support to training programs for students that enhance self-efficacy and strengthen their intrinsic motivation and metacognitive strategies. In one word, the study supported and expanded SRL of the mathematics area in the theoretical implication, and provided many SRL training skills for the mathematics education in the practical implication.

In spite of its value, the study has certain limitations which need to be overcome. First, there are different assessment of metacognition, such as self-report questionnaires, think-aloud protocols, and systematic observation of behavior (Desoete, 2008). The self-report used by the current study is subjective and is vulnerable to false memory or cognition bias (Veenman, 2011). It should be better to utilize logfile to measure metacognitive regulation in the future research (Winne, 2010). Second, the hypothesis model was only explored in the mathematics course, and we should investigated the model of several courses to verify the stability and diversity. Third, the measurement of MK of self (difficulty/lack of fluency) and MK of tasks in MKMQ may be not suitable for Chinese students. So the generalization of our findings to western educational context is still an open question as the huge difference between China and western world on math education. Finally, since the sample is not sex-balanced, it is not suitable to test the factor invariance of the relationships among the variables in the model. Future studies should increase the sample size and balancing sex as much as possible for comparison. Meanwhile, it is valuable in the future to model the role of many other variables based on our framework (such as metacognitive experience or skills, anxiety, attitude, and time) to reveal the full picture of effective math learning.

## Conclusion

The present study demonstrated that metacognitive knowledge exerted its effect on mathematics performance through the indirect path via the sequential mediating effect of self-efficacy and intrinsic motivation. The findings add to the growing literature by highlighting the underlying mechanism by which MK contributes to mathematics performance.

## Ethics Statement

The current research was consistent with the ethical principles of human subjects, and had been approved by “The ethics board of Faculty of Psychology, Beijing Normal University.” First, we told the detailed content of the study to the teachers, students, and their parents. Second, participants and their parents signed the informed consent on a voluntary basis.

## Author Contributions

The study was designed by JL. YT and YF collected the data. All authors analyzed the data and wrote the paper.

## Conflict of Interest Statement

The authors declare that the research was conducted in the absence of any commercial or financial relationships that could be construed as a potential conflict of interest.
